# Stent Placement and Coil Embolization for Plastic Bronchitis in a Patient With Hypoplastic Left Heart Syndrome

**DOI:** 10.7759/cureus.80824

**Published:** 2025-03-19

**Authors:** Fumiya Inoue, Shin Ono, Hideaki Ueda

**Affiliations:** 1 Pediatric Cardiology, Kanagawa Children's Medical Center, Yokohama, JPN

**Keywords:** cardiovascular stent, congenital heart disease (chd), fontan operation, hypoplastic left heart syndrome (hlhs), plastic bronchitis

## Abstract

Plastic bronchitis is a severe complication following the Fontan procedure, characterized by the formation of tracheal casts, leading to respiratory failure. No established treatment exists for post-surgical plastic bronchitis. We present the case of a three-year-old boy with hypoplastic left heart syndrome who developed plastic bronchitis two years after the Fontan procedure. Despite initial treatments, his condition did not improve until catheterization was performed to place a stent in the left pulmonary artery and embolize aortopulmonary collateral vessels. This combined approach resulted in significant clinical improvement and no recurrence until 17 months. Elevated pulmonary venous pressure can lead to increased mucin secretion from the tracheal epithelium; reducing this pressure may be effective for treating plastic bronchitis. Our case demonstrates the successful application of simultaneous stenting and coil embolization, resulting in a rapid decrease in pulmonary venous pressure and an improvement in the patient's condition. This combined approach may offer a promising therapeutic option for managing plastic bronchitis after the Fontan procedure.

## Introduction

Plastic bronchitis is a potentially fatal complication that can occur after the Fontan procedure, which causes the formation of casts in the trachea, leading to respiratory failure. Two types of bronchitis are reported: inflammatory (type 1) and surgical (type 2) [1]; anti-inflammatory therapy, such as steroid treatment, is considered effective in treating the former; however, no treatment has been established for the latter. After the Fontan procedure, 4-14% of patients may develop plastic bronchitis [2], and the mortality rate of plastic bronchiolitis after congenital heart disease surgery is as high as 14-50% [3]. Previous reports demonstrated cases in which stenting alone improved the outcome [4,5] and those in which embolization of collateral vessels tended to improve the outcome [6]. In the present study, we performed both procedures simultaneously to treat a patient with hypoplastic left heart syndrome and unbalanced pulmonary blood flow presenting with plastic bronchitis.

## Case presentation

**Figure 1:**
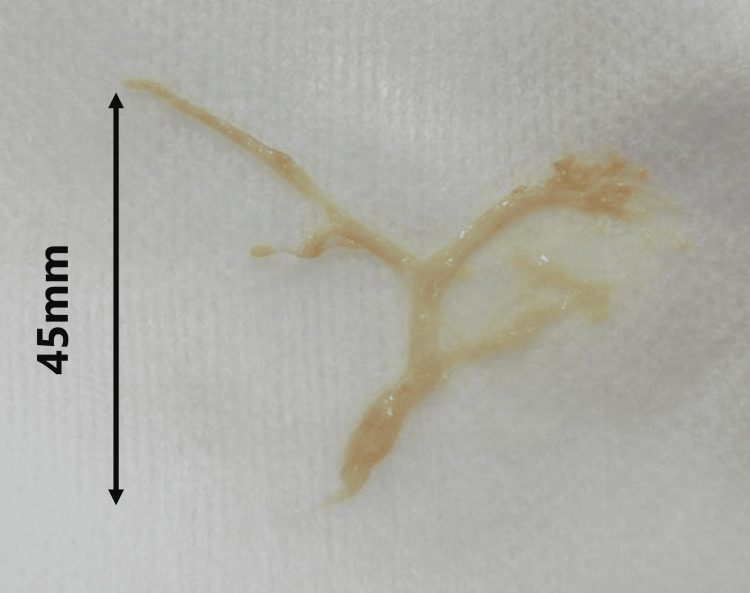
The cast expectorated by the patient measures 45 mm in length

**Figure 2:**
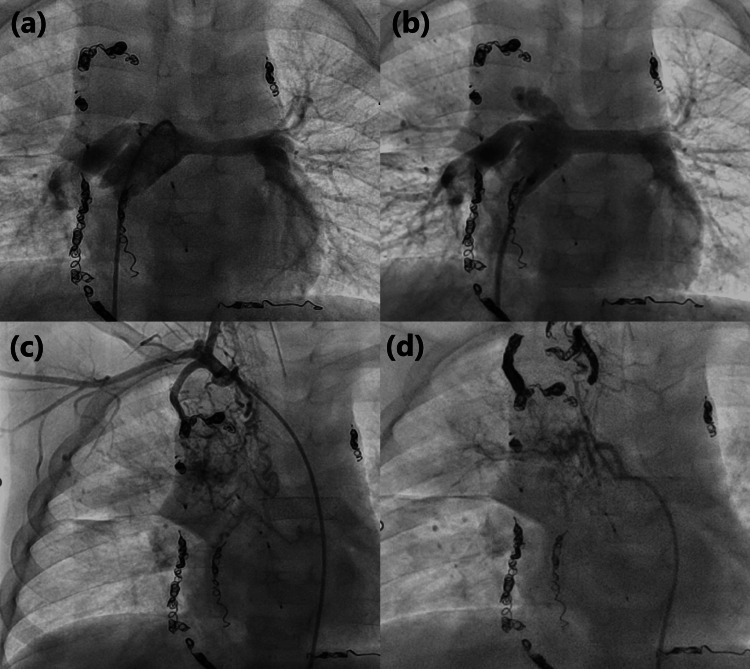
Angiograms (a) Left pulmonary artery angiogram of the patient before and (b) after stent implantation, showing a double diameter. (c) Right subclavian artery angiogram and (d) right bronchial artery angiogram, demonstrating many tiny aortopulmonary collateral vessels. The other visible coils and plugs were placed prior to the Fontan procedure.

A three-year-old boy, weighing 2,717 g, was born at 38 weeks and two days of gestation. He was diagnosed with hypoplastic left heart syndrome (mitral and aortic valve atresia) at birth, consistent with the fetal diagnosis. Bilateral pulmonary artery banding and balloon atrial septostomy were performed at two and 28 days of age, respectively. The ductus arteriosus was maintained with lipo-prostaglandin analogs, simultaneous Norwood and bilateral Glenn procedures were performed at four months of age, and balloon angioplasty for postoperative left pulmonary artery stenosis was performed at five months. The azygos vein had been ligated, but recanalization was confirmed by a previous angiogram. Therefore, it was closed using plugs (Amplatzer™ Vascular Plug 4; Abbott Medical, Plymouth, MN) at seven months of age. Two months prior to the Fontan procedure, embolization of the aortopulmonary collateral arteries was performed using coils (Interlock®; Boston Scientific, MA). The patient underwent the fenestrated Fontan procedure despite a difference in the caliber of the left and right pulmonary arteries (left 4.2 mm, right 8 mm; left pulmonary artery to conduit pressure gradient, 2 mmHg) at the time of pre-Fontan evaluation catheterization. In the early postoperative period following the Fontan procedure, right chylothorax was observed, and it improved with the use of octreotide and a fat-restricted diet. The fenestration was spontaneously closed three months postoperatively. The patient developed a cough two years postoperatively. Sudden expectoration of casts (Figure 1), along with decreased oxygenation, was observed, and the patient was re-admitted to the hospital. On admission, his saturation of percutaneous oxygen level was approximately 80% under oxygenation therapy using a high-flow nasal cannula (30 L/min, fraction of inspired oxygen level 0.7); coarse crackles were heard in the right upper lung field; and facial edema, indurated leg edema, and hepatomegaly were observed. Serum blood tests revealed hypoalbuminemia (3.0 g/dL) and hypoproteinemia (5.5 g/dL). Chest X-ray revealed extensive infiltrates, primarily in the right upper lung field. Contrast-enhanced CT showed a 3.5-mm stenosis extending from the main pulmonary artery to the left pulmonary artery. The patient was treated with respiratory support and physical therapy for expectoration, increased tadalafil dose to lower the pulmonary artery pressure, and a lipid-restricted diet with albumin supplementation. These treatments were ineffective; therefore, on the seventh day of hospitalization, catheterization was performed to place a stent in the main pulmonary artery (Figure 2a). The left pulmonary artery pressure averaged 11 mmHg, the conduit pressure averaged 15 mmHg, the pressure gradient between them was 4 mmHg, and the narrowest point was 3.6 mm against a reference vessel diameter of 6.4 mm. Before the stent placement, we used an 8-mm × 20-mm Mustang™ Balloon Dilatation Catheter (Boston Scientific, MA) and performed angiography of the original aorta to confirm no compression of the coronary arteries extending from the original aorta. An 8-mm × 27-mm Express Vascular® LD (Boston Scientific, MA) was implanted. Subsequently, the diameter of the narrowest point of the left pulmonary artery increased to 7.2 mm, which was detected via angiography (Figure 2b), and the pressure gradient was reduced to 2 mmHg. Because the right lung was considered the site of cast formation and abundant aortopulmonary collateral vessels extending to the right lung field were detected, those aortopulmonary collateral vessels from the right subclavian (Figure 2c) and bronchial (Figure 2d) arteries were embolized with coils (Interlock®; Boston Scientific, MA). The patient was discharged 10 days after catheterization and did not experience expectoration of cast sputum. After discharge, there has been no recurrence for 17 months.

## Discussion

Elevated pulmonary venous pressure can lead to increased mucin secretion from the tracheal epithelium [6]; treatment to reduce pulmonary venous pressure may be effective for plastic bronchitis. To the best of our knowledge, no previously reported treatment included both stenting and coil embolization procedures, which have been performed simultaneously in the present study. This approach helped correct the difference in pulmonary blood flow between the right and left lungs and reduce cardiac workload, resulting in a rapid decrease in the right pulmonary venous pressure on the diseased side [7]. There have been reports of cases in which patients with plastic bronchitis were improved by creating a fenestration [7]. If the patient does not respond to this treatment, reopening the fenestration may be considered as an option with the expectation of further reduction in pulmonary venous pressure.

In addition, this patient had a history of chylothorax after the Fontan procedure, and lymphatic damage from the operation was suspected, which is a common factor in plastic bronchiolitis [8]. Balloon angioplasty was performed immediately after the Glenn operation because of severe left pulmonary artery stenosis; hypoplasia of the left pulmonary artery was also observed on catheter evaluation before the Fontan procedure. This may have led to an increase in right pulmonary artery blood flow and right pulmonary static blood flow on the diseased side. Furthermore, a considerably increased blood flow in the right somatic collateral pulmonary vessels led to a direct increase in pulmonary venous pressure that may have contributed to cast formation. Various treatments have been proposed for plastic bronchitis, including steroid administration, N-acetylcysteine inhalation, bronchodilators, urokinase, tissue plasminogen activator inhalation, antibiotics, and a high-protein, low-fat diet; however, no established treatment method has been found [8].

## Conclusions

In this study, we report a case of plastic bronchitis developed in a three-year-old boy two years after undergoing a Fontan procedure. The condition was successfully managed by simultaneously stenting the main pulmonary artery and performing coil embolization of the aortopulmonary collateral arteries. We suggest that aggressive hemodynamic correction may lead to a better outcome in plastic bronchitis after the Fontan procedure. Larger case studies would be required to establish the efficacy of simultaneous stenting and embolization in treating this condition.
